# Double trouble: synchronous extrahepatic cholangiocarcinoma and gallbladder cancer in a Caucasian woman with no pancreaticobiliary maljunction

**DOI:** 10.1093/jscr/rjab587

**Published:** 2022-01-21

**Authors:** Fraser Hugh Simpson, Michael Auld, Harsh Kandpal, Kayla Tran, Manju D Chandrasegaram

## Abstract

Synchronous malignancies of the bile duct and the gallbladder are rare. These cases are often associated with pancreaticobiliary maljunction which is characterized by a long common shared pancreatobiliary channel leading to the Sphincter of Oddi. This predisposes the biliary epithelium to pancreatic enzyme reflux and makes the development of neoplasia more likely. We describe the case of a 64-year-old Caucasian female who presented with new jaundice and severe cholecystitis secondary to an impacted gallstone which was seen on ultrasound. Magnetic resonance cholangiopancreatography was organized with suspicion of a possible Mirizzi syndrome. This revealed a mid-distal bile duct cancer in addition to cholecystitis from an impacted gallstone. She was treated with intravenous antibiotics for her cholecystitis and underwent an urgent endoscopic retrograde cholangiopancreatography procedure for biliary decompression and stenting for her obstructive jaundice. The patient proceeded to pancreaticoduodenectomy with final histopathology revealing a synchronous primary gallbladder malignancy in addition to the known bile duct cancer.

## INTRODUCTION

Synchronous cancers of the extrahepatic bile duct and the gallbladder are rare [1]. Synchronous cancers can occur as two separate and distinct primary tumours or from metastatic spread of an original cancer to a different site [2]. Synchronous gallbladder and extrahepatic cholangiocarcinoma (EHC) are often associated with an abnormal pancreatobiliary duct junction (APBDJ) [3, 4].

As synchronous cancers can occur in the absence of APBDJ, other causes of synchronous cancers of the biliary tree have been suggested [5]. These include the primary tumour metastasizing and becoming multifocal, local extension of a primary cancer or other anatomical variants which are undetectable with current investigation [6].

Most cases of synchronous cancers are reported from Japan or India. A Japanese case series demonstrated that 5–7.4% of patients with an EHC had a separate gallbladder cancer [7–9]. There is a lack of similar worldwide data which may suggest geographical or other causal factors.

Herein we present the case of a 64-year-old woman from Australia who presented with cholecystitis and was discovered to have a coexistent EHC. She went on to have a pancreaticoduodenectomy which revealed an incidental synchronous gallbladder adenocarcinoma in addition to her bile duct cancer.

## CASE REPORT

A 64-year-old Caucasian female presented with a 4-day history of increasing right upper quadrant pain, nausea and anorexia. Her past medical history included a previous caesarean section, open appendicectomy, laparoscopic hysterectomy and a 25 pack-year smoking history. On examination she was visibly jaundiced, was tender in the right upper quadrant and had a positive Murphy’s sign. Serum biochemistry revealed deranged liver enzymes. She was treated for acute cholecystitis.

**Figure 1:**
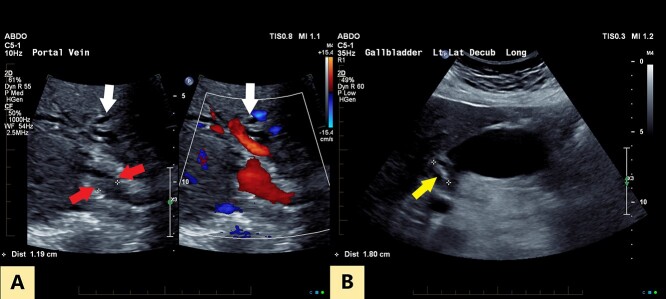
(**A**) Transverse oblique US reveals segmental wall thickening of the common hepatic duct (between red arrows) with intrahepatic duct dilatation (white arrows). (**B**) Distended gall bladder with calculus in the neck (yellow arrow).

**Figure 2:**
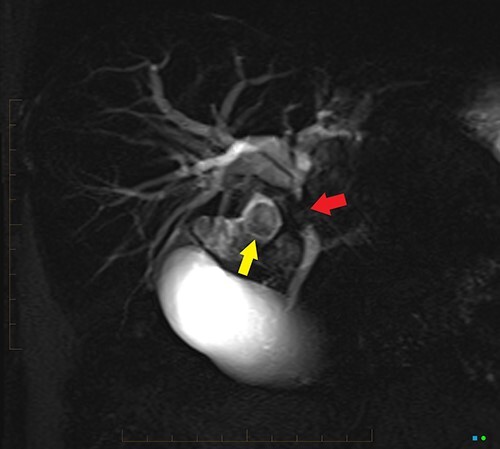
2D projectional MRCP reveals abrupt segmental stricture (red arrow) of the common hepatic duct with marked intrahepatic biliary dilation. Gall bladder is distended and there is a calculus (yellow arrow) in the gall bladder neck.

**Figure 3:**
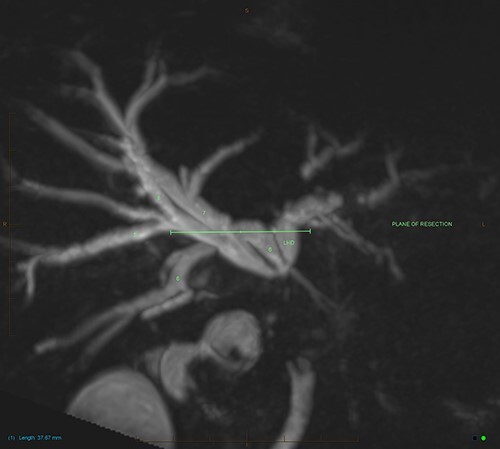
MIP reconstruction of 3D MRCP reveals abrupt segmental stricture of the common hepatic duct with marked intrahepatic biliary dilation. Trifurcation of the biliary confluence into the left hepatic duct, aberrant segment 6 duct and the right hepatic duct. The right hepatic duct is formed by the union of right anterior (segment 5,8) duct and segment 7 duct while the segment 6 duct is joining separately at the confluence. The approximate plane of surgical resection is indicated.

An ultrasound of the abdomen revealed a large gallstone in the gallbladder neck measuring 36 mm with mild biliary dilatation (Fig. 1). Magnetic resonance cholangiopancreatography (MRCP) revealed an irregular long stricture of the extrahepatic bile duct beginning distal to the primary biliary confluence, involving the common hepatic duct for a distance of 20 mm (Fig. 2). A multiphase liver magnetic resonance imaging was performed which confirmed a malignant appearing stricture of the common hepatic duct and showed an ill-defined soft tissue lesion in the porta hepatis between the gallbladder neck and the common hepatic duct (Fig. 3). Her Ca 19.9 was 160 kU/L (ref range < 35 kU/L).

An endoscopic retrograde cholangiopancreatography procedure (ERCP) confirmed a likely malignant biliary stricture in the extrahepatic duct and a plastic biliary stent was placed for biliary decompression. An FDG-PET scan revealed intense FDG uptake in the extrahepatic bile duct, with mild FDG uptake in two lymph nodes surrounding the gallbladder neck with no distant metastasis. Multidisciplinary consensus was obtained to proceed to surgery in the setting of likely EHC.

**Figure 4:**
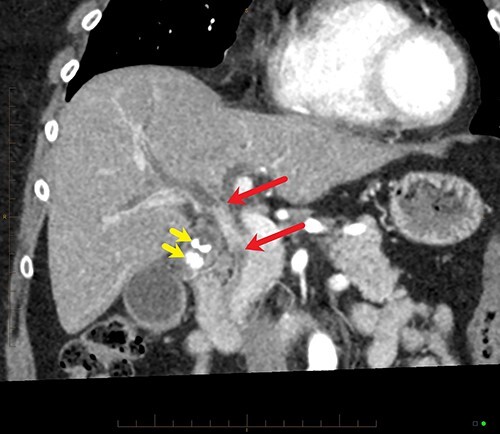
Coronal MPR of late arterial phase CT reveals segmental common hepatic duct stricture with circumferential wall thickening and enhancement (between red arrows) resulting in intrahepatic duct dilatation. Radiopaque calculi (yellow arrows) in the gallbladder.

The patient underwent an open pancreaticoduodenectomy and cholecystectomy. Surgical resection was extended proximally to include the biliary confluence and biliary transection was performed to reveal four separate bile duct openings consisting of the right anterior and posterior sectoral ducts, a separate segment 6 duct and the left hepatic duct (Fig. 4).

**Figure 5:**
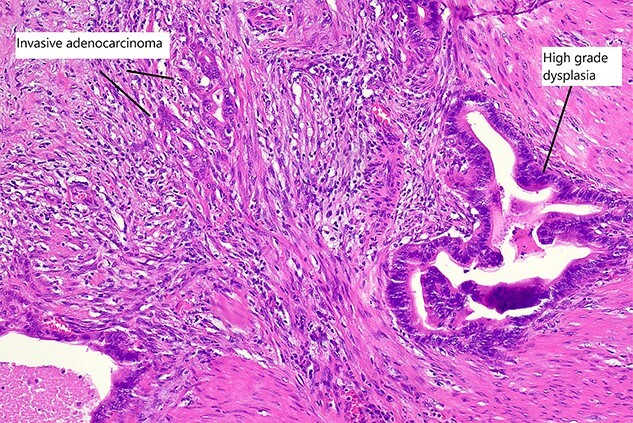
High magnification slide showing high-grade dysplasia and invasive adenocarcinoma of the gallbladder after haematoxylin and eosin staining.

Final histopathological assessment of the resected specimen reported a common bile duct adenocarcinoma (cholangiocarcinoma) measuring 37 mm in greatest dimension with extensive perineural, periductal, perinodal and peripancreatic soft tissue involvement. Metastatic carcinoma was found in 3/17 lymph nodes resected. The cholangiocarcinoma was staged as pT2N1M0. The gallbladder was found to have extensive high-grade biliary intraepithelial neoplasia with a primary gallbladder adenocarcinoma measuring 18 mm in greatest dimension entirely separate from the cholangiocarcinoma (Fig. 5). The patient recovered well from the procedure and was discharged home from hospital on post-operative Day 15. She has proceeded to have further treatment with adjuvant chemotherapy.

## DISCUSSION

Synchronous malignancies are known to occur throughout the gastrointestinal tract. Gertsch, Thomas, Baer, Lerut, Zimmermann and Blumgart [2] have established criteria to define true synchronous malignancies of the biliary tract. Firstly, there should be no direct continuity between the tumours, and secondly that the growth patterns are typical of each primary tumour and lastly that there are clear histologic differences between the two tumours [2]. In the case presented the tumours are not continuous, each growth pattern is typical of the respective cancers, and histologically the two tumours are different.

Synchronous cholangiocarcinoma and gallbladder cancers are thought to arise when the bile duct and gallbladder are both affected by carcinogenic alterations of the affected cells. This increases the likelihood of synchronous cancers as both cholangiocarcinoma and gallbladder cancer arise by the metaplasia–dysplasia-carcinoma sequence in the presence of chronic inflammation [10]. This process is known as field cancerization and different stimuli are known to contribute to the presence of multiple carcinomas in the biliary tree [11].

Reports describing synchronous biliary tract cancer are rare and according to Fujii, Kaneko, Sugimoto, Okochi, Inoue, Takeda, et al. [12] 62.5% of synchronous cancers of the biliary tract are associated with an APBDJ. It is speculated that this anatomical abnormality predisposes the bile duct and gallbladder to exposure to pancreatic enzymes [10]. This is thought to lead to chronic inflammation of the entire biliary tree driving carcinogenesis [12].

Outside of Japan and India, one case series was published in Switzerland detailing four cases of synchronous EHC and gallbladder cancer [2]. It is thought that Japan has a high incidence of synchronous cholangiocarcinoma and gallbladder cancer due to its high incidence of APBDJs. Studies have shown the incidence of APBDJ to be 1:1000 in Japan compared to 1:100 000 in the Western world [13].

Additional proposed risk factors for gallbladder cancer and cholangiocarcinoma include female gender, gallstone disease, smoking, bacterial injury from Salmonella species or Helicobacter species and exposure to heavy metal poisoning [14]. Our patient did have a significant smoking history, gallstones on presentation and was female, but history did not reveal any overt exposure to carcinogenic substances or bacteria.

## CONCLUSION

Although exceedingly rare, synchronous cholangiocarcinoma and gallbladder cancers do occur in Western populations with few risk factors. It is important that cases of synchronous biliary malignancies in populations outside of India and Japan are published to highlight the possibility of synchronous disease in these populations. Additionally, it is important that a thorough examination of the gallbladder is performed after pancreaticoduodenectomy for malignancy to ensure that there is no additional gallbladder cancer.
